# Addressing perinatal depression in a group of underserved urban women: a focus group study

**DOI:** 10.1186/1471-2393-14-336

**Published:** 2014-09-26

**Authors:** Nancy C Raymond, Rebekah J Pratt, Amy Godecker, Patricia A Harrison, Helen Kim, Jesse Kuendig, Jennifer M O’Brien

**Affiliations:** Deborah E Powell Center for Women’s Health, Departments of Psychiatry and Family Medicine and Community Health, Medical School, University of Minnesota Medical School, MMC 293 Mayo, 420 Delaware Street SE, Minneapolis, MN 55454 USA; Family Med/Community Health, MMC 381 Mayo, 420 Delaware St SE, Minneapolis, MN 55455 USA; Carbone Cancer Center, University of Wisconsin-Madison, 370 WARF Building, 610 Walnut Street, Madison, WI 53726 USA; Minneapolis Health Department, Director of Research and Program Development, 250 4th St. S., Room 510, Minneapolis, MN 55415-1384 USA; Hennepin Women’s Mental Health Program, 914 South 8th Street, Suite S-110, Minneapolis, MN 55404 USA

**Keywords:** Perinatal, Mental health, Qualitative data, Focus group

## Abstract

**Background:**

Perinatal mental health problems are common complications of pregnancy that can go undetected and untreated. Research indicated that mental health complications are more prevalent in women from disadvantaged communities, yet women from these communities often experience barriers to accessing treatments and interventions. Untreated depression during pregnancy can lead to poor self-care, increased substance abuse, poor obstetrical outcomes, developmental delay in children, and increased risk of postpartum depression. In this study we investigated the perceived perinatal mental health needs of our participants and they wanted to address their perceived needs.

**Methods:**

In this qualitative study, we invited women who resided in an underserved, urban community who were pregnant or who delivered within the past year to participate in focus groups.

**Results:**

Thirty-seven women participated in seven focus groups. Thirteen themes emerged which were described in relation to mental health needs, help currently accessed and the type of support wanted. The themes included the various mental health needs including dealing with changing moods, depression, feelings of isolation, worrying and a sense of being burdened. Women described using a limited range of supports and help. Participants expressed a preference for mental health support that was empowerment focused in its orientation, including peer support. Women also described the compounding effect that social and economic stresses had on their mental health.

**Conclusions:**

Participants wanted access to a greater range of supports for mental health than were currently available to them, including peer support, and wanted assistance in addressing social and economic needs. These findings offer a challenge to further broaden the types of services offered to women, and demonstrate that those services need to be responsive to the challenging contexts of women’s lives. Integrating women’s views and experiences into the development of services may help to overcome barriers to care.

## Background

Perinatal depression is widely reported to be the leading complication of childbirth [1]. Prevalence rates of depression in pregnant women have been estimated at between 6.5% to 12.9% [2]. As many as 19.2% of women have a depressive episode during the first 3 months postpartum [2]. Despite this level of need, the utilization of perinatal mental health services is low in terms of both accessing treatment and continuing treatment [3]. Untreated depression during pregnancy can lead to poor self-care, increased substance abuse [4], poor obstetrical outcomes [5], developmental delay in children [6, 7], and increased risk of postpartum depression [8]. Despite its widespread prevalence, perinatal depression frequently goes undetected and disproportionately impacts disadvantaged women and their children. Access to help for perinatal conditions is also influenced by socioeconomic factors, with disparities negatively impacting perinatal mental health [9].

Despite the extent of perinatal mental health problems, much can be done to address mental health needs for women in the perinatal period. Cognitive behavioral therapy (CBT) has been shown to improve depression [10]. Additionally, there is evidence that CBT depression treatments can be culturally adapted to improve their effectiveness with cultural minorities and low socioeconomic groups [11, 12]. CBT and pharmacological interventions may offer effective treatment, but can increase costs of services [13], which may be a barrier for providing care [4–8]. Other approaches have also been explored, such as interpersonal psychotherapy [14, 15], mindfulness [16] and the use of postpartum doulas [17]. The location of treatment delivery has also been researched, and shows that the delivery of evidence-based treatments by home visiting nurses can also be effective at addressing postpartum depression [18–21].

While the evidence base for effective treatment is growing, women are still not consistently accessing services for perinatal mental health problems. Research indicates that only a minority of women who are at risk for perinatal depression will receive referrals to address mental health issues, and fewer yet will ultimately engage in treatment [22]. The literature reports barriers to accessing help including a lack of knowledge or understanding about perinatal mental health issues [23], along with previous negative experiences with health care providers or a fear of being judged by health care providers [23]. Women have described how they value being able to talk with peers about their experiences [24]. Some studies indicate that accessing peer support was also potentially seen as the only acceptable option, due to negative views or experiences with more formal services [24, 25]. Stigma related to accessing mental health services has also been identified as an important barrier for accessing help [26].

Despite existing research, there is a need to further our understanding of what kind of mental health needs women think they have, and what kind of support or help they would be likely to access to address those perceived needs. Integrating women’s views and experiences into the development of services may help to overcome some of the barriers such as lack of trust and negative perceptions about accessing help [25]. This research focused on the geographic area of Minneapolis, Minnesota, a racially and culturally diverse large urban center with a population of over 380,000 [27]. Almost one-quarter of Minneapolis families with children under age five live in poverty.

The investigators conducted a needs assessment to inform future development of public health systems approaches to reduce negative outcomes due to perinatal depression, which we defined as the time a woman conceives through the first year post-partum. Here we report on the findings from the focus groups conducted with women in the Minneapolis area as part of that needs assessment. The focus groups were specifically interested in exploring the following research objectives:

A) What perceived needs do women describe they have in relation to their mental health through the perinatal period?
B) What help do women describe current seeking in relation to addressing mental health concerns during the perinatal period?
C) What support do women describe wanting for addressing mental health concerns during the perinatal period?

## Methods

Women who were pregnant, or who delivered within the past year, were recruited to participate in focus groups intended to gain information on the mental health needs of women during the perinatal period. Women were recruited from three health care clinics, which were located in disadvantaged parts of the city and serving traditionally underserved populations. Staff in the clinic recruited women into the study from the population of women receiving prenatal or postnatal care in their clinic. Focus groups were conducted at the clinics from which the women were recruited. Groups were combined discussions of women receiving both prenatal and postnatal care, and focused on exploring a breadth of experiences.

### Participants

Thirty-seven women participated in the focus groups. Participants came from diverse backgrounds. Seven of the participants were non-Hispanic Caucasian (19%), 11 of the participants were African American (31%), two of the participants were American Indian (6%), three identified as multiracial (8%), and 14 of the participants were Latina or Hispanic (36%). Twenty-one women (57%) were born in the US, 12 (32%) in Mexico; one in Somalia and one in Ecuador. The average age of the women who participated was 27.5. Thirty-three women said they had a partner or spouse (89%), and twenty three (70%) of those women were living with that partner or spouse. Twenty one (56%) of the women were pregnant at the first focus group.

### Procedures

Institutional Review Boards (IRB) of the University of Minnesota and Hennepin County Medical Center reviewed and approved this project as exempt for review. The study adhered to the RATS guidelines on qualitative research [28].

A moderator’s guide was created with input from project stakeholders, which included members of the research team and community partners working in the area of perinatal mental health. The questions fell within the following categories: feelings and experiences of women after having a baby; how depression is viewed by these women and their families; help seeking behavior they have engaged in; current coping strategies used to deal with mental health challenges; and preferred types of mental health services they would like to access (format, features, location and time). The questions were semi-structured, and the focus group facilitators encouraged participants to share their views and experiences on these topics and interact with each other during the discussion.

Participants were recruited from three sites by case managers at a federally qualified health care center in North Minneapolis and by prenatal care providers at Hennepin County Medical Center and a county clinic in south Minneapolis. Prospective participants were provided with a brochure describing the study and contact information for one of the two study coordinators. Recruits who were interested signed a release of information form to permit a study coordinator to contact them, or contacted the study coordinator directly. During the telephone conversation, the study coordinator verbally described the study and presented each element of the consent form. If the recruits chose to participate, they signed up for a specific focus group session at the end of the call.

Seven focus groups were conducted with three to eight women in each group. Focus groups were conducted by Minnesota Department of Health staff who had specifically been trained in focus group facilitation and had experience conducting focus groups. Five groups were conducted in English (25 women) and two groups were conducted in Spanish (12 women). The average age of the women participating in the focus groups was 27.5. At the beginning of each session, the facilitator went through the informed consent process with all of the participants, allowed time for questions, and obtained written consent from each participant. Participants were asked to complete a brief questionnaire, designed by the research team, describing socio-demographic characteristics and were told that this information would not be connected with their consent forms, in order to protect their confidentiality.

All focus groups were recorded and transcribed verbatim by a professional transcription service. The groups that were conducted in Spanish were transcribed in Spanish and then translated to English by professional translators. Each transcript was systematically reviewed for themes. This entailed one member of the research team (RP), working through each transcript, with the assistance of NVivo9 (QSR), and coding themes as they arose in the data. A series of themes and subthemes in the text were identified. The analysis was informed by a social constructivist version of grounded theory [29, 30], where the researchers allow themes to emerge from the data, but acknowledge the influnce of additional sources on the analytic process, such as current literature, approach to the research, or knowledge on the topic. The research team met on two occasions to review the emerging codes alongside the data, and discuss any areas differences in interpretation until consensus was reached on the themes. Additionally the researchers discussed a summary of the findings with the research participants to further validate the emerging analysis. This discussion took place in the context of presenting key findings for further review and discussion at an additional focus group held with the same participants.

## Results

The themes which emerged from the analysis are presented in relation to each of the research questions, namely a description of perinatal health needs, the current perception of available mental health resources, and women’s desire for greater mental health supportive services. Here we discuss each of these main areas and their associated sub-themes, which are summarized in Table 1.

**Table 1:** **Focus group themes and sub-themes**

Research question	Sub-themes
Perinatal mental health needs	Pregnancy was viewed as a time of experiencing mental health challenges
	Mental health concerns were exacerbated by life circumstances
	There are a range of emotional and mood challenges in the perinatal period
	Social and emotional context impacts mental health
Mental health help currently available	Reliance on informal support
	Reliance on self
	Accessing services
Mental health help wanted	Mental health needs where not sufficiently met
	Outreach services were considered important
	Focusing on empowerment oriented support
	Peer support
	Educational based
	Additional services

### Perinatal mental health needs

#### Pregnancy was viewed as a time of experiencing mental health challenges

Participants described a range of mental health needs, including dealing with changing moods, depression, isolation, worry and a sense of burden. Focus group participants described these needs as relating to pregnancy as a time of dealing with new and changing moods, hormonal changes and the challenges of life transitions. Some described living in a constant state of irritation.

*It’s just a constant thing, it’s all day every day with me. It’s when I’m hungry, when I’m sleepy, when I’m tired, I just get irritated for – I don’t even – I get irritated for no reason sometimes. (Focus Group 6)*

### Mental health concerns were exacerbated by life circumstances

Some women described how some variations in mood were seen to be problematic and proceeded difficulties such as depression. Challenging life circumstances were seen by some as contributing to variations in mood and depression. For some this was associated with the challenges presented by the life transitions arising from having a baby, including disruptions to work or schooling. Focus group participants also identified that isolation and loneliness were serious issues facing women through the perinatal period. Some participants described feeling very alone, in general, with limited or no friend or family networks. For others this was an issue that was worsened by having an unsupportive, or absent, significant other.

*And you just feel alone. I mean like I have three older children and they have a dad and you know, he has a different dad, … this dude I mean he’s not there, we don’t communicate, it’s no communication, none whatsoever. So it’s just harder all around the board. (Focus Group 1)*

### There are a range of emotional and mood challenges in the perinatal period

A range of other emotional issues emerged from the focus group discussion. While less prominent than the prospect of dealing with normal changes in moods which may grow into depression, other needs included dealing with feelings of anger, frustration, loss, grief, regret, anxiety and addressing the emotional impact of birth trauma.

### Social and emotional context impacts mental health

The other prominent themes that were described as impacting mental health were economic and situational, rather than emotional. Many women in the focus groups expressed a persistent sense of worry about how to move through the life transition of having a baby, and all the responsibilities of providing for children. This was exacerbated by limited support. Many focus group participants were approaching parenthood with a great sense of burden about providing for their family, including the potential loss of income during pregnancy and after the child was born.

*I’m on the verge of getting put on bed rest because this is my fourth child but he’s bigger than all my other children and it’s like basically if I get put on bed rest – I only work part time. That’s going to be a lot on me, worried about paying my bills and taking care of my other children. So I mean it’s not just all about eating healthy and worrying about gaining weight, it’s a lot. (Focus Group 1)*

Overall, the needs expressed illustrate that social and economic needs, such as difficulties with housing, and financial worries, along with emotional needs were all seen as having an impact upon mental health. This is particularly the case in circumstances in which women have limited or no support for facing these challenges.

### Mental health help currently available

Women in the focus groups were asked to describe what they currently do to address their mental health needs, and they described a wide range of resources they access.

### Reliance on informal support

Despite the social and economic barriers to accessing mental health services, participants exclusively focused on how they met their emotional needs. One prominent approach was to rely on informal networks, such as friends and family. Many participants described the importance of having people to talk to about pregnancy issues. Partners were seen as a valuable resource if the relationship was supportive; however many women did not have such a relationship to rely on, and drew on networks of friends for support instead. Participants described the benefits of sharing mutual experiences, and the usefulness of talking with someone who can empathize, someone who has shared the same, or similar, experience. This general support and advice was seen as a way to address mental health needs.

*I usually go to my mom or my sister, like my sister she’s older than me and she’s got three kids, this will be my first baby. So I go to women who have had kids before. That’s where usually I go. This my first baby so I usually just take advice from women who has already been there. (Focus Group 6)*

### Reliance on self

Many participants described relying mostly on themselves to get through difficult times. This included using strategies such as journaling. A certain degree of relying on the self was seen as important for identifying how to address one’s mental health needs.

*Yeah, figuring it out by yourself is good too because ain’t nobody else gonna sit here and have the answer for you, only you know. You know? (Focus Group 1)*

### Accessing services

Some focus group participants had used therapy from a range of different professionals, to help address their mental health needs. Counseling and therapy was seen as a useful way to get support and identify strategies for coping. Some focus group participants used medications for depression and anxiety. This seemed to be particularly the case where there was a pre-existing issue with depression, or a previous experience of perinatal mood disorders.

Aside from the main areas of mental health support, there was lesser mention of a very broad range of ways to address mental health needs, such as using acupuncture, going to church, using a doula, support from a hospital, peer support group, psychiatrist, midwife and social worker.

### Mental health support wanted

Women in the study described the various forms of help or support they wanted to be able to access to address their mental health needs.

### Mental health needs where not sufficiently met

Participants expressed a desire for a range of mental health support that was more comprehensive than the range of services that were currently available, indicating that there was a level of unmet need for this group. One key theme emerging from the analysis indicated that women wanted help that was accessible, including not being turned away for lack of health insurance and meeting childcare needs.

### Outreach services were considered important

Women wanted the mental health support to include outreach to women in the community as a way to reach women who were having difficulties and to overcome access issues. For some women, they felt that home visits would also be important. Women described having a hard time seeking help, or even leaving the house, if they were suffering from depression. Home visits were seen as a proactive outreach approach to identifying women with mental health problems who may be reluctant to leave their home.

*I think it [home visits] would be helpful, …because when I’m depressed I’m not going out, I’m sitting on the couch. And somebody to come in and see what’s going right and do I have what I need and am I really set up for success? (Focus Group 3)*

### Focusing on empowerment oriented support

Women expressed the need to have mental health support that focused on supporting and empowering them to find solutions in a way that would build on their strengths and encourage resilience. One such approach that was seen as highly desirable by the focus group participants was peer support. They described the peer support as potentially being provided by other mothers, of all ages, including support from older women who had been through similar challenges. The prospect of being able to talk with and get support from someone who had been through a similar experience was seen as valuable, in part because that support would be seen as having credibility.

*I mean it’s possible for that to work out for you, but it ain’t for everybody to be honest, I mean like they really don’t understand what you just went through. It’s gotta be experience, you can’t just say it and then you understand – how you gonna understand somebody what they just said? You can only understand why they are going through what they just said. But you can’t sit here and say I know what you mean. What you mean? Were you there when I did all this? You need to be in my shoes to really know that, you know what I’m saying? (Focus Group 1)*

### Peer support

The value of peer support was evident as it was frequently used within the group sessions themselves, as participants shared their experiences and offered encouragement to each other. One participant gave an example of how she encourages other women.

*Yeah just being yourself and getting past it, trying to put it behind you, look forward, don’t look back. If you look back you just gonna keep getting worse –that’s why I tell people if something happen today then oh well, keep it pushing. The past is the past and the past and look to new beginnings, looking forward to new beginnings and new outcomes. (Focus Group 6)*

### Educational approach

The prospect of mental health support being delivered in an educational format was described as being very desirable. Educational settings (a class or course) could be used to address broad issues such as preparation for pregnancy and childbirth, and early parenting. The setting was seen as an accessible way to bring together groups of parents to access support that would enhance mental health. Educational settings offered an advantage because some women felt it might more productively engage their partners in discussion and learning about perinatal mental health.

### Increased services

Participants also described a range of specific services they thought should be offered such as being able to access a crisis phone line, faith-based support services, and assistance from doulas. There was a request for mental health services that could address broad social or economic difficulties by offering financial advice, housing assistance, and transport services.

## Discussion

This study was designed to talk with women about what mental health needs they feel they have, what help they currently access and what help they would like to address their mental health needs. These needs include those that address emotional needs and ways to alleviate social and economic stressors that have a negative impact on their mental health. Women in the focus groups were asked to describe what they currently do to address their mental health needs. They primarily described relying on support from friends or family or their own strengths, although some acknowledged the usefulness of medication or therapy. This is consistent with other studies that have indicated women may turn to informal supports to meet their needs [24]. However the literature is mixed on the effectiveness of some forms of peer support for postpartum depression [31], although there is some growing indication that it may be an effective approach [17]. Women in this study were quite adamant that peer support would be a preferred option for mental health support. This may indicate that further work needs to be done to review the quality and nature of the peer support interventions offered in the perinatal field, as research on the use of peer support in general mental health indicates it can both be effective and very well received by those seeking mental health improvement [32, 33].

Some women talked about the use of formal treatments, and this remains an important option. However our findings echoed other literature in highlighting the need for service providers to be cognizant of the fact that many women may have had negative experiences previously, and thus have negative perceptions of services [24, 25]. Yet there remains a strong field of research about the usefulness of formal treatments [10], including their ability to be culturally adapted for use [11, 12], and more work needs to be done to address the barriers women face in accessing services.

A clear implication of this work is that the women want those helping them to address their mental health by using an empowerment orientated approach. They also preferred the use of an educational format. These findings highlight that a barrier to engaging women with current services might originate in the approach to services themselves, which use a medical model instead of an empowerment oriented approach to address mental health needs.

One particularly notable finding in this research was how strongly women believed that social and economic challenges, such as difficult relationships, lack of adequate housing, financial issues or unemployment, contribute to mental health problems. Yet when women talked about what they currently do to address mental health needs, all actions focused on addressing emotional needs. Equally, the literature reflects this, as most interventions also focus on emotional needs [10–13, 16, 17]. There are some exceptions in the literature, with some interventions focusing on multiple risk factors [34], but these approaches appear to be primarily focused on improving health outcomes, rather than overall needs which may contribute to poor mental well-being. This signals the potential importance of addressing social and economic needs into services as one potential way to increase the perception of relevance and accessibility for women.

### Limitations

This focus group study used a small group of women from one geographical area, and they may have self-selected into the study based on their interest and experience with mental health issues. This means there are limits to how broadly these findings can be generalized. Additionally we did not screen women for mental health problems, or ask them to disclose their experiences with mental health services, and this may limit generalizability of our findings.

## Conclusion

Women in our study stated that they wanted access to a greater range of different kinds of support for mental health problems, such as educational groups or peer support. They also described significant social and economic needs which were rarely addressed in current services. These findings offer a challenge to further broaden the types of services offered to women to include peer support, resilience focused programming, and educational programming. Also, in order to increase relevance and accessibility for diverse women, mental health services should more clearly address social and economic challenges.

## Abbreviations

CBT: Cognitive behavioral therapy
IRB: Institutional review boards.
